# A Generalized Beta Model for the Age Distribution of Cancers: Application to Pancreatic and Kidney Cancer

**DOI:** 10.4137/cin.s3050

**Published:** 2009-08-04

**Authors:** Tengiz Mdzinarishvili, Michael X. Gleason, Leo Kinarsky, Simon Sherman

**Affiliations:** Eppley Cancer Institute, University of Nebraska Medical Center, 986805 Nebraska Medical Center, Omaha, Nebraska, 68198-6805. Email: ssherm@unmc.edu

**Keywords:** cancer, incidence rates, aging, histopathology

## Abstract

The relationships between cancer incidence rates and the age of patients at cancer diagnosis are a quantitative basis for modeling age distributions of cancer. The obtained model parameters are needed to build rigorous statistical and biological models of cancer development. In this work, a new mathematical model, called the Generalized Beta (GB) model is proposed. Confidence intervals for parameters of this model are derived from a regression analysis. The GB model was used to approximate the incidence rates of the first primary, microscopically confirmed cases of pancreatic cancer (PC) and kidney cancer (KC) that served as a test bed for the proposed approach. The use of the GB model allowed us to determine analytical functions that provide an excellent fit for the observed incidence rates for PC and KC in white males and females. We make the case that the cancer incidence rates can be characterized by a unique set of model parameters (such as an overall cancer rate, and the degree of increase and decrease of cancer incidence rates). Our results suggest that the proposed approach significantly expands possibilities and improves the performance of existing mathematical models and will be very useful for modeling carcinogenic processes characteristic of cancers. To better understand the biological plausibility behind the aforementioned model parameters, detailed molecular, cellular, and tissue-specific mechanisms underlying the development of each type of cancer require further investigation. The model parameters that can be assessed by the proposed approach will complement and challenge future biomedical and epidemiological studies.

## Introduction

The number of newly diagnosed primary cancers at particular organ sites occurring in a specified population during a given time period (for instance, one year or five years), is called the cancer incidence rate. The rate of cancer incidence for a specific age group during this time period is called the age-specific incidence rate. Usually, the age-specific incidence rates are presented as the number of cancers per 100,000 persons in a specified age group. The sequence of age-specific incidence rates for all specified age groups is referred to as the age distribution of a given cancer. A mathematical modeling of the age distribution of cancer results in a simple analytical function, *I*(*t*), that can approximate observed values of cancer incidence rates and provides parameters of this function. The obtained model parameters can be further used to build rigorous statistical and/or biological models of cancer development.

Development of mathematical models of age-specific cancer incidence rates began more than 55 years ago. Analyzing cancer mortality rates in the UK, Nordling,^1^ as well as Armitage and Doll,^2^ noticed the existence of two age periods in which cancer mortality manifests differently. In the initial age period, a number of cancer mortalities per population at a given age is equal or close to 0. For the majority of adult onset cancers, this period is extended between birth and an age when the cancer presentation begins growing exponentially. In the second age period, the cancer mortality per population at a given age is exponentially growing with aging.

The first mathematical model of cancer presentation in aging was proposed by Armitage and Doll (the AD model).^2^ This model can be presented in the following way:

(1) $$I(t)=ct^{k-1}$$

where *I*(*t*) is the modeled cancer incidence rate at age *t; c* is a parameter characterizing overall cancer susceptibility in a population at cancer risk, and *k* is the number of stages of cancer development. This model describes the relationship between cancer incidence rates and aging, when cancer development is in the exponential growth phase.

Cook, Doll and Fellingham found a single increasing linear trend for the logarithm of many cancer incidences plotted as a function of the logarithm of age at diagnosis,^3^ presumably reflecting accumulated lifetime carcinogenic risks and/or exposures. This led to conclusions that: (i) the number of stages of cancer presentation (parameter *k*) can vary between different cancer tissues, but is constant for a given cancer organ site, and (ii) the overall cancer susceptibility (parameter *c*) may be dependent on the geographical location (country of residency) of the population at cancer risk.

Extrapolation of the AD model to ages older than 70 years (up to which observed data was considered reliable at the time the AD model was proposed) can lead to a statement that *if a person lives long enough, sooner or later he/she will get cancer*. However, Cook, Doll and Fellingham considered the possibility of flattening incidence rates of cancer in ages above 60.^3^ For this purpose, they assumed that only a very limited and fixed fraction of the whole population is susceptible to a particular type of cancer. In this case, the cancer-sensitive fraction of a population will decrease with increasing age that, in turn, will cause a flattening of the cancer incidence rates at old ages.

In the second half of the 20th century, the quality of the collected cancer incidence rate data has improved markedly. Thanks to the implementation of the Surveillance, Epidemiology, and End Results (SEER) program, a lot of reliable data on the cancer incidence rates for specific organ sites at different ages (including the oldest ones) were collected.^4^ Using the SEER database, Pompei and Wilson showed that patterns of the age-specific incidence rate for the fixed time period (2000–2004) for most common adult cancers have a turnover point (near the age of 80),^5^ after which these patterns have a tendency to fall and may reach a value of 0 as age increases toward the end of the human life span. This observation encouraged Pompei and Wilson to extrapolate the AD model beyond the age of 70 to the life span by adding an additional term to Equation 1, resulting in:

(2) $$I(t)={\left(at\right)}^{k-1}(1-bt)$$

where the *a* parameter is a constant for limiting stage transitions; *k* reflects the number of these rate-limiting (slow) stages required to initiate cancer; and *b* is a parameter, whose meaning can be easily described by its reciprocal value, 1/*b*, that presents an age at which *I*(*t*) becomes 0. Equation 2 is a special form of the Beta function.

Recently, Harding, Pompei, Lee and Wilson modified Equation 2 to:

(3) $$I(t)=ct^{k-1}(1-bt)$$

where the *c* parameter characterizes a combined rate constant for limiting stage transitions. Values of the *c*, *k* and *b* parameters can be determined by best-fitting the age-specific incidence rates collected in the SEER database.^6^ Below, we will refer to this model as the Pompei and Wilson (PW) model.

By adjusting *c*, *k* and *b* of Equation 3, Harding and coauthors performed curve fitting for the age-specific cancer incidence rates for 20 major organ sites listed in SEER for males and 21 major organ sites listed for females.^6^ A satisfactory data fitting was shown for many of the examined cancer sites. It was also shown that the age-specific incidence rate distributions demonstrated a common shape. In 36 of the 41 considered cancer sites (for males and females), this common shape was characterized by the location of the corresponding distribution peaks (the incidence rate turnover near the age of 80), and relatively small (<10%) variability of 1*/b* (near age of 100). The *k* values varied between 2.4 and 10.6. Very large variations (more than five orders of magnitude) were found for the *c* values.

In all the aforementioned works, the modeling of age distribution of cancer was performed without considering time period and cohort effects. However, ignorance of these effects could seriously distort cancer presentation in aging.^7^–^10^ Recently, Meza, Jihyoun, Moolgavkar, and Luebeck carried out an adjustment of the observed age-specific incidence rates of colorectal and pancreatic cancers for birth cohort and time period effects and did not observe a turnover point at old ages.^11^ These authors proposed to present distribution of the adjusted cancer incidence rates by a composition of two analytical functions: (i) a power (or exponential) function that up to the age of approximately 60 years remains the same as in the AD model; and (ii) a linear function, after the ages of 60+. Thus, Meza and coauthors not only rejected the existence of the turnover points of the age distribution of cancer at old ages (at least in the cases of the colorectal and pancreatic cancers) but also stated that for the ages beyond 60 years the linear function approximates the adjusted observational data better than other functions used before.

In addition to ignorance of the time period and cohort effects, there are other potential problems in the utilization of mathematical models for studies of the age distribution of cancer. Most models use raw incidence rate data without omitting cases corresponding to second primary or secondary tumors, and do not omit cases which have not been microscopically confirmed. Also, these models do not provide means to calculate confidence intervals for the determined model parameters.^12^

The present work is aimed at overcoming the aforementioned shortcomings in the modeling of age distribution in cancer.

## Materials and Methods

### Data preparation and filtration

To build mathematical models for age distribution in PC and KC, we used data from the SEER 9 registries that contain cancer data collected in the following nine locations: Atlanta, Connecticut, Detroit, Hawaii, Iowa, New Mexico, San Francisco-Oakland, Seattle-Puget Sound, and Utah. In the SEER database, each case record contains information on whether this is the first primary malignant case and whether the case is histopathologically confirmed. Limiting inclusion to cases where the patient was of known race and whose case indicated a first primary, microscopically confirmed tumor is considered to be a filtered data, and data where this filtering was not performed is considered to be a raw data. For age distribution modeling, we used the filtered data, which are expected to be more reliable than raw data. We utilized the incidence rate data expressed per 100,000 persons to the nearest 0.0001 decimal place and age-adjusted by the direct method to the 2000 United States standard population.^13^

We used SEER 9 data collected during the 20-year time period between 1985 and 2004. To smooth out random fluctuations, the data were combined in four five-year cross-sectional time periods: 1985–1989; 1990–1994; 1995–1999; and 2000–2004. For PC and KC, the gender-specific incidence rates were grouped into 18 five-year age groups: 17 groups, ranging from 0 to 84 years old, and the 18th group that included all cases for ages 85 or over. For each of these intervals, *i*, the corresponding *I*(*t_i_*) and standard errors (*SE_i_*) were obtained by processing the SEER data according to SEER’s rate algorithms.^14^ For each age interval, the values of the coefficient of variance were also determined as: *CV_i_* *= SE_i_* */ I*(*t_i_)*

### Birth cohort and time period adjustments

We assumed that each observed incidence rate, *I_ij_*(*t_i_*), can be estimated as a product of the corresponding coefficient of time period effect, *v_j_*, coefficient of cohort effect, *u_l_*, and theoretical incidence rate (or the hazard function depending only on age), *h*(*t_i_*), i.e.

(4) $$I_{ij}(t_{i})=v_{j}u_{l}h(t_{i})$$

where *i*, *j*, and *l* are indexes of the age, time period and birth cohorts, correspondingly; and *t* = *t_i_* is the midpoint of the corresponding age group.^11^,^15^ Indices *i* and *j* determine index *l* (see below). The birth cohort and time period adjustments performed in this work can be easily described by the use of Table 1 and Table 2.

Table 1 schematically presents the incidence rate of data collected in 1985–1989, 1990–1994, 1995–1999, and 2000–2004. In this Table, the incidence rates of the same cohorts are located along diagonals. We used data for the age groups over age 30 (index *i* = 7, …, 18), because the incidence rates for these age groups are statistically different from 0. We also limited our analysis by nine birth cohorts (index *l* = 1, …, 9). The first cohort includes patients that were born in years of 1915–1919, while the ninth cohort is formed from patients born in 1955–1959. In Table 1, each of these nine birth cohorts are marked by an arrow linking the diagonal cells, in which the cancer incidence rates observed for this group in each time period are presented.

Table 2 schematically presents the observed incidence rates as a product of the hazard function, *h*(*t_i_*), and the corresponding time period and birth cohort coefficients, *v* and *u*. As can be seen on the corresponding diagonals in Table 2, there are four approximations of the observations related to the first cohort: *v*_1_*u*_1_*h*(*t*_15_), *v*_2_*u*_1_*h*(*t*_16_), *v*_3_*u*_1_*h*(*t*_17_), *v*_4_*u*_1_*h*(*t*_18_). Analogously, there are four approximations for each of the other eight cohorts. (Note that in this Table we did not provide data corresponding to other cohorts: the cells that should be assigned for the 1900–04, 1905–09, 1910–14, 1960–64, 1965–69, and 1970–74 birth cohorts are empty. For these cohorts, the numbers of observations are less than four and their corresponding coefficients, *u*, should be treated with a lower weight than ones for the considered groups. Therefore, we used the most homogeneous and reliable data.)

From Table 2, the relationship between indexes *i*, *j*, and *l* can be presented as: *l* = *j − i* + 15. Now, assuming the absence of the cohort effect (*u* = 1) and using Equation 4 and Table 2, we can estimate the ratios of the coefficients of the time period effect in the following way:

(5) $$\frac{v_{1}}{v_{2}}=\frac{1}{8}\sum_{i=8}^{15}\frac{I_{i,1}}{I_{i,2}};\;\;\;\frac{v_{2}}{v_{3}}=\frac{1}{8}\sum_{i=9}^{16}\frac{I_{i,2}}{I_{i,3}};\;\;\;\frac{v_{3}}{v_{4}}=\frac{1}{8}\sum_{i=10}^{17}\frac{I_{i,3}}{I_{i,4}}.$$

From this system of three Equations we can obtain thee unknowns, *v*_2_, *v*_3_, and *v*_4_, by setting *v*_1_ = 1.

Analogously, assuming the absence of the time effect (*v* = 1), we can obtain estimates of the ratios of the coefficients of the cohort effect:

(6) $$\begin{array}{ll} \frac{u_{1}}{u_{2}}=\frac{1}{3}\left(\frac{I_{15,1}}{I_{15,2}}+\frac{I_{16,2}}{I_{16,3}}+\frac{I_{17,3}}{I_{17,4}}\right); & \frac{u_{2}}{u_{3}}=\frac{1}{3}\left(\frac{I_{14,1}}{I_{14,2}}+\frac{I_{15,2}}{I_{15,3}}+\frac{I_{16,3}}{I_{16,4}}\right);\\ \frac{u_{3}}{u_{4}}=\frac{1}{3}\left(\frac{I_{13,1}}{I_{13,2}}+\frac{I_{14,2}}{I_{14,3}}+\frac{I_{15,3}}{I_{15,4}}\right); & \frac{u_{4}}{u_{5}}=\frac{1}{3}\left(\frac{I_{12,1}}{I_{12,2}}+\frac{I_{13,2}}{I_{13,3}}+\frac{I_{14,3}}{I_{14,4}}\right);\\ \frac{u_{5}}{u_{6}}=\frac{1}{3}\left(\frac{I_{11,1}}{I_{11,2}}+\frac{I_{12,2}}{I_{12,3}}+\frac{I_{13,3}}{I_{13,4}}\right); & \frac{u_{6}}{u_{7}}=\frac{1}{3}\left(\frac{I_{10,1}}{I_{10,2}}+\frac{I_{11,2}}{I_{11,3}}+\frac{I_{12,3}}{I_{12,4}}\right);\\ \frac{u_{7}}{u_{8}}=\frac{1}{3}\left(\frac{I_{9,1}}{I_{9,2}}+\frac{I_{10,2}}{I_{10,3}}+\frac{I_{11,3}}{I_{11,4}}\right); & \frac{u_{8}}{u_{9}}=\frac{1}{3}\left(\frac{I_{8,1}}{I_{8,2}}+\frac{I_{9,2}}{I_{9,3}}+\frac{I_{10,3}}{I_{10,4}}\right). \end{array}$$

By setting *u*_1_ = 1, we can find eight unknowns, *u*_2_, *u*_2_, …, *u*_9_, from this system of eight Equations.

When the exact mathematical form of the hazard function *h*(*t*) is unknown, we experience the well-known “identifiable problem.” In this case, simultaneous evaluation of the time period and cohort effects can only be performed using additional assumptions.^8^ To solve this problem, we used an iterative technique proposed by Luebeck and Moolgavkar.^15^

Initially, we assumed that the cohort effect was absent (*u* = 1) and evaluated coefficients of the time period effect, *v*, using the system of equations (Equation 5). Then, we fixed the obtained time period coefficients and corrected the observed incidence rates by dividing them by the coefficients presented in Equation 4. Continuing, we estimated the coefficients of the cohort effect, *u*, from the system of equations (Equation 6), in which the time effect-corrected incidence rates were used. Assuming *v* = 1 in Equation 4 and using the estimated cohort effect coefficients *u*, the incidence rates can be corrected one more time.

This adjusting procedure aims to correct possible systematic errors in the observed age-specific incidence rates, *I_ij_*. After such an adjustment, the incidence rates mainly contain random errors that can be treated by standard statistical approaches. In the calculations presented below, we used age-specific incidence rates adjusted for time period and cohort effects.

### Generalized beta model

To fit the filtered observational data on age-specific incidence rates, we tested various models, such as: a gamma function, a Weibull function, a special variant of the Beta function proposed by Harding and coauthors,^6^ and the Generalized Beta (GB) probability distribution function defined as:

(7) $$Ir(T)=c{\left(bT\right)}^{k-1}{\left(1-bT\right)}^{m-1}$$

where *T* = (*t − A*), *t* is the age at cancer diagnosis; the incidence rate *Ir* (*T* ) = *I*(*t*); *b* = 1/ (*B − A*);

*A* and *B* are the lower and upper age limits of cancer development, respectively; *c* is a generalized rate constant; *k* − 1 and *m* − 1 are the degrees of increase and decrease in cancer incidence rates, correspondingly.

Our results suggest that the best fit can be obtained by using the GB function presented by Equation 7. *I*(*t*) can be also presented as:

(8) $$I(t)=c{\left(\frac{t-A}{B-A}\right)}^{k-1}{\left(\frac{B-t}{B-A}\right)}^{m-1}$$

For each age interval, *j*, the corresponding age-adjusted incidence rates, *I*(*t_j_*), and their standard errors (*SE_j_*), can be obtained from the SEER data; and the coefficient of variance, *CV*, can be determined as: *CV_j_* = *SE_j_* / *I*(*t_j_*)

For each age interval, the SEER data presents the incidence rates as well as the number of cases, which can be considered as a Poisson distribution.^10^,^14^ For large case numbers, the incidence rates can be viewed as variables that are approximately normally distributed around expected *I*(*t_j_*) with standard error *SE_j_*. To calculate incidence rates, we considered only those age intervals that contain at least five cases; otherwise we assumed that in the corresponding age interval the value of the incidence rate was 0.

Values of the *A* and *B* age limits of cancer development can be considered as known *a priori*. Traditionally, the lower age limit *A* has been chosen as 0, assuming that the process of cancer development starts from the birth.^3^ The upper age limit, *B*, can be treated as an approximation of the upper limit of the life span, or the age at which the best curve-fitting is obtained.^5^,^6^ It should be noted that the model variables *c, k* and *m* are very sensitive to variations of the *A* and *B* age limits. In this case, the problem of the curve-fitting becomes a so-called “ill posed” problem. Therefore, the use of *a priori* information is necessary to stabilize the solution against variations of the input parameters, *A* and *B*.^16^ In this work, for simplicity, we fixed the age interval of cancer development as: *A* = 0 year and *B* = 100 years.

Thus, for each age interval, *i*, one can calculate *T_i_* = (*t_i_* − *A*) and obtain *Ir* (*T_i_*) = *I*(*t_j_*) and their standard errors *SE*[*Ir*(*T_i_*)] = *SE_i_*. Taking logarithms from both sides of Equation 7 in each age interval, one can obtain a system of linear Equations:

(9) $$\begin{array}{l} \text{ln}\;Ir(T_{i})=\text{ln}\;c+(k-1)\text{ln}\;bT_{i}\\ +(m-1)\text{ln}(1-bT_{i}),\;i=1,2,\ldots ,n. \end{array}$$

Where *n* is the number of the considered age intervals.

According to Equation 8, the system (Equation 9) can be rewritten as:

(10) $$\begin{array}{l} \text{ln}\;I(t_{i})=\text{ln}\;c+(k-1)\text{ln}\;\left(\frac{t_{i}-A}{B-A}\right)\\ +(m-1)\text{ln}\;\left(\frac{B-t_{i}}{B-A}\right),\;\;\;i=1,2,\ldots ,n. \end{array}$$

Three unknown parameters, ln *c*, (*k* − *1*) and (*m* − *1*), can be determined from Equation 9 or 10 by minimizing the following function R* using a weighted least square method:

(11) $$\text{min}\;R^{*}=\sum_{i=1}^{n}w_{i}{\left(O_{i}-C_{i}\right)}^{2},$$

where *w_i_* is a weight of the *i*-th residual, (*O_i_* − *C_i_*), which is the deviation between the observed value, *O_i_*, of the ln [*Ir*(*T_i_*)] and its expected value calculated by Equation 9 in the following way:

(12) $$\begin{array}{l} C_{i}=\text{ln}\;c+(k-1)\text{ln}\;bT_{i}+(m-1)\text{ln}\;(1-bT_{i})\\ =\text{ln}\;c+(k-1)\text{ln}\;\left(\frac{t_{i}-A}{B-A}\right)+(m-1)\text{ln}\;\left(\frac{B-t_{i}}{B-A}\right) \end{array}$$

Based on the rules of the error propagation,^17^ one can show that the variance of errors for ln [*Ir*(*T_i_*)] is approximately equal to the square of the coefficient of a variation of the incidence rate: *CV_i_*^2^ = (*SE_i_* / *I*(*t_i_*)^2^. It also can be shown that, when *w_i_* = (1/*CV_i_*)^2^, the weighted sum of residuals

(13) $${R^{*}}_{\nu }=R^{*}=\sum_{i=1}^{n}w_{i}{\left(O_{i}-C_{i}\right)}^{2}$$

has a *χ*^2^*_ν_* distribution with *ν* = *n* − *p* degrees of freedom, and *p* = 3 is the number of derived parameters.^18^

By numerical experiments, we have shown that for small variances of error in incidence rate data, the distribution of errors of ln *Ir* (*T_i_*) is close to normal. On the other hand, systems like systems (Equations 9 and 10) with normally distributed errors in the dependent variable, can be solved by multiple linear regression analysis.^19^ Therefore, in this work we used the multiple linear regression analysis to solve the system (Equation 10). To estimate the goodness of model fitting, we used a standard *χ*^2^ test.^19^

## Results and Discussion

### Comparison of distributions of the raw and filtered age-specific incidence rates for PC and KC

**Figure 1:**
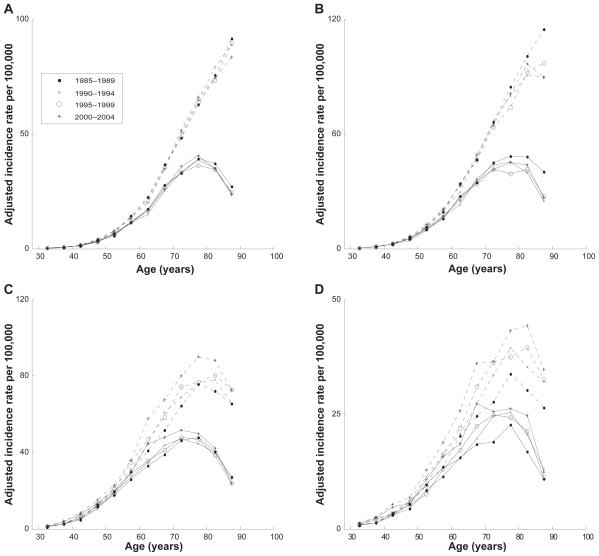
**Age-specific incidence rates of PC and KC obtained from the raw and filtered SEER data.** (**A**) PC in white males; (**B**) PC in white females; (**C**) KC in white males; (**D**) KC in white females. **Notes:** Incidence rates obtained from the raw and filtered data are shown as dashed and solid lines, respectively. Time periods of data presented in panel **A** are shown in the legend; time periods presented in panels **B**, **C**, and **D** are the same as in panel **A**.

As described in *Materials and Methods*, we extracted the raw and filtered age-specific incidence rates for PC and KC for white males and females collected in the SEER 9 database during the years of 1985–2004 and combined these data in four time period subsets: 1985–1989, 1990–1994, 1995–1999, and 2000–2004. The age distributions of the raw and filtered data, gathered in each of these subsets, are shown in Figure 1. As can be seen from this figure, the age patterns corresponding to the raw and filtered incidence rates for the same type of cancer have significantly different amplitudes and shapes. The large differences in amplitudes are caused by the inclusion of cases of non-first primary and metastatic cancer, as well as cases with microscopically unconfirmed tumors in the raw data. The number of cases excluded by these criteria are detailed in Table 3. In addition, the age patterns of the filtered incidence rates exhibit the existence of a decline at old ages, while in the cases of unfiltered data this fall is not evident. The obvious deceleration/decline that the filtered incidence rates exhibit at age 75 and over cannot be caused just by a diagnostic bias at old ages, but rather it strongly suggests an influence of basic biological processes on carcinogenesis and the rates of clinical cancer manifestation at an old age.^20^ Because it is clear that the filtered incidence rates represent more reliable and more homogeneous statistical data than the raw data, in the present work we exclusively used the filtered data.

**Figure 2:**
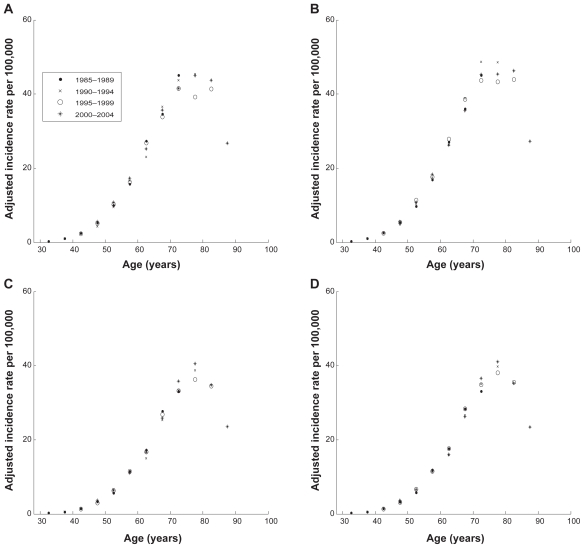
**Age-specific incidence rates for PC in white males and females obtained from the filtered SEER data.** (**A**) Unadjusted for time period and cohort effects incidence rates for white males; (**B**) adjusted for time period and cohort effects incidence rates for white males; (**C**) unadjusted for time period and cohort effects incidence rates for white females; (**D**) adjusted for time period and cohort effects incidence rates for white females. **Notes:** Time periods of data presented in panel **A** are shown in the legend. Time periods presented in panels **B**, **C** and **D** are the same as in panel **A**.

**Figure 3:**
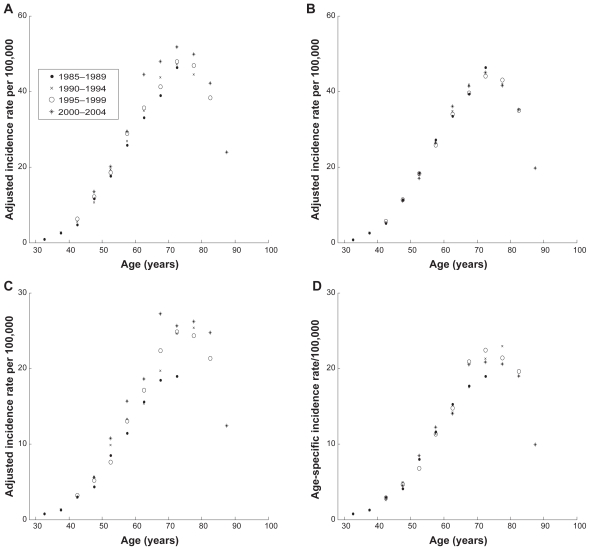
**Age-specific incidence rates for KC in white males and females obtained from the filtered SEER data.** (**A**) Unadjusted for time period and cohort effects incidence rates for white males; (**B**) adjusted for time period and cohort effects incidence rates for white males; (**C**) unadjusted for time period and cohort effects incidence rates for white females; (**D**) adjusted for time period and cohort effects incidence rates for white females. **Notes:** Time periods of data presented in panel **A** are shown in the legend. Time periods presented in panels **B**, **C** and **D** are the same as in panel **A**.

Figures 2 and 3 show how time period and cohort adjustments affect the overall shape of age distributions of PC and KC for white males and females. Because all adjustments were made by using data schematically shown in Table 2, these figures presented the age distributions of nine cohorts (1915–1919–1920–1924–1925–1929–1930–1934–1935–1939–1940–1944–1945–1949–1950–1954; and 1955–1959) during four time periods (1985–1989–1990–1994–1995–1999; and 2000–2004). Therefore, during the first considered time period, 1985–1989, these nine cohorts exhibit incidence rates in the following nine five-year age intervals: 30–34; 35–39; 40–44; 45–49; 50–54; 55–59; 60–65; 65–69; and 70–74. During the next considered time periods, these nine cohorts exhibit incidence rates in the nine five-year age intervals shifted by five years compared to the previous time period.

Thus, for the 30–34 age interval, only one observation made in the first time period, 1980–1984, was used. For the 35–39 age interval, two observations made in the first and second time periods, 1980–1984 and 1985–89, correspondingly, were used. Analogously, three observations for the age interval 40–44 made in the first three time periods and four observations for each of the 50–54, 55–59, 60–64, 65–69 and 70–74 age intervals, made during all considered time periods were utilized. In the cases of the 75–79, 80–84, and 85+ age intervals, three, two and one observations were used as shown in Table 2.

Figures 2 and 3 show influence of adjustments on PC and KC incidence rates, correspondingly. Below, we demonstrate that the age distributions of the adjusted incidence rates for PC and KC can be very well approximated using the generalized Beta function defined by Equation 7.

## Mathematical models of age distribution of PC and KC

To estimate the model parameters in Equation 8, we minimized the weighted sum R* (Equation 11) by a least squares method. To do this, we utilized the *regress* function of the MATLAB software package.^21^ For this function, we used *n* = 35 values of the adjusted incidence rates *I_ij_*(*t_i_*), and their *SE_i_* as input data. The rate for the 85 + age interval was not used due to an uncertainty of its middle point position. This resulted in three estimated model parameters, *c*, *k*, and *m*, and their 95% confidence intervals (CI), assuming that *A* = 0 and *B* = 100. The obtained parameters are supposed to determine the best curve fitting that system (Equation 10) can provide for the adjusted incidence rates that were used as input data.

We examined the goodness of the curve fit by the *χ*^2^ test for the values of the weighted sum of residuals R*_ν_ (Equation 13). The degrees of freedom, *ν* = *n* − *p*, was defined by the number of used data points (*n* = 35), less the number of derived parameters ( *p* = 3). According to the standard *χ*^2^ test with the 0.05 significance level, if the value of R*_ν_ was outside the interval (*χ*^2^_0.025,_*_ν_*; *χ*^2^_0.975,_*_ν_*), we would reject the hypothesis that our model fits the observed data. The two tail limit values of the *χ*^2^ test were *χ*^2^_0.025,32_ = 18.3 and *χ*^2^_0.975,32_ = 49.5, correspondingly. Therefore, in the case when the weighted sum of residuals, R*_ν_, is within the interval 18.3 < R*_ν_ < 49.5, one can conclude that the null hypothesis (that the distribution of the modeled incidence rates obtained by Equation 10 fits the set of adjusted values) is not rejected at the 0.05 significance level.

**Figure 4:**
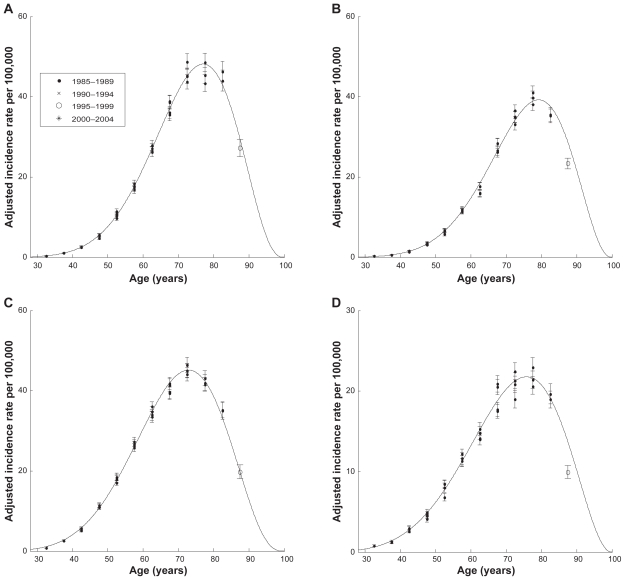
**The GB approximation of the age-specific incidence rates adjusted for time period and cohort effects**. (**A**) PC in white males; (**B**) PC in white females; (**C**) KC in white males; (**D**) KC in white females. **Note:** Error bars denote standard errors (SE).

Table 4 presents the obtained values of model parameters and descriptive statistics. Figure 4 shows the results of modeling of the age-specific incidence rates of PC and KC for the white male and female populations. As was mentioned previously, the incidence rates for the age interval 85+ were not used as input data for the curve fitting. These incidence rates are shown on Figure 4 only for illustration purposes. Figure 4 shows that the modeled incidence rates well approximate the adjusted values of the observed incidence rates (including the 85+ point). This good visual fit is strongly supported by the *χ*^2^ tests, which suggest that in all cases the values of the weighted sum of residuals, R*_ν_ , are within the given interval, 18.3 < R*_ν_ < 49.5 (see Table 4).

In Table 4 the parameters *k* and *m* assess the degrees of the increase and decrease in cancer incidence rate, correspondingly. The parameter *c* is related to an overall risk of getting a cancer for a given population. The point of inflection indicates the point in which the second derivative of *I*(*t*) (Equation 7) is equal to 0, which corresponds to the age at which the decrease begins to prevail over the increase in cancer incidence rate. We did not consider the second point of inflection due to uncertainties of data at very old ages. The maximum indicates the age at which the incidence rate reaches its maximum, after which the cancer incidence rate declines.

Table 4 and Figures 4A and 4B suggest that the parameter *c* for PC in white males is higher than for white females. However, the confidence intervals of this parameter for males and females are slightly overlapping. For PC, the values of the parameter *k* in males and females are statistically indistinguishable, while the values of parameter *m* for males are statistically higher than for females. The higher value of *m* in males is a result of the point (age) of inflection and maximum incidence rate, which occur about two years earlier in males than in females. This may suggest that biological mechanisms of PC development differ in white males and females.

As can be seen from Table 4 and Figures 4C and 4D, in the case of KC all three model parameters, *c*, *k*, and *m*, for white males are statistically higher than those for white females. The point of inflection for males appears two years earlier than that for females, while the maximum of cancer incidence rate for males appears about three years earlier than that for females. Notable differences in all parameters that characterize the age distributions of KC in males and females may suggest distinct biological mechanisms of KC development in white males and females.

A comparative analysis of the age patterns of the PC and KC incidence rates (see Table 4) suggests that for these types of cancer, the value of parameter *m* is greater than 2. This is in contrast to the assumption made by Harding and coauthors,^6^ where it was postulated that for all types of cancers, *m* should be equal to 2. As for parameter *k*, for PC in males and females the values of *k* are statistically higher than the ones for KC, which resulted in an earlier presentation of inflection points for KC incidence rates than for the PC (see Table 4). Analogous comparisons show that the peaks of the KC incidence rates appear four years earlier than the corresponding ones of the PC. These comparisons suggest the existence of distinct organ-specific biological mechanisms of the carcinogenesis in the pancreas and kidney.

### Goodness of curve fitting for different model functions

**Figure 5:**
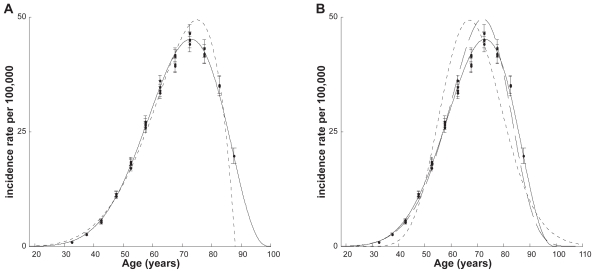
**Comparison of goodness-of-fit of the KC incidence rates in white males performed by the GB model (solid line) and other models. (A**) The GB model vs. the PW model (dotted line). (**B**) The GB model *vs.* models described by the Gamma (dotted line) and Weibull (dashed line) functions. **Note:** Error bars denote standard errors (SE).

Among existing models,^2^,^3^,^5^,^6^,^11^,^15^ only special types of the Beta function as proposed by Pompei and Wilson,^5^ and Harding and coauthors,^6^ have an ability to describe the turnover of incidence rates at old age (for other models, incidence rates are monotonically increasing). There are other well-known statistical models, such as the Gamma and Weibull functions, which, in principle, can also be used to describe this turnover. Therefore, using the weighted least squares method, we compared the goodness-of-fit of our proposed GB function with the goodness-of-fit of the special type of the Beta function (PW model), as well as the Gamma and Weibull functions. Figure 5A shows the comparison of fitting of observational KC data for white males using the GB model versus the PW model. Figure 5B shows an analogous comparison of the GB, Gamma, and Weibull functions.

As can be seen from Figure 5, the GB model fits the pattern of the KC incidence rates in white males much better than the other considered models, and this visual appearance is well supported by the use of *χ*^2^ statistic. In fact, the data from Table 4 show that the observed incidence rates of KC in white males can be very well fitted by the GB model. However, for the PW model and the Gamma and Weibull functions, the standard *χ*^2^ test rejects the hypotheses that the curves described by these functions fit the observed data with the 0.05 significance level. In fact, for the PW model, the value of this statistic is equal to 565.0 with *ν=* 32 degrees of freedom (where *ν= n − p*, *n =* 35 – the number of used data points, and *p =* 3 − the number of parameters to be derived). Analogously, for the Gamma function (*ν =* 33, two estimated parameters) this statistic is equal to 1,720.6, and for the Weibull function this statistic is equal to 208.0 (*ν =* 33, two estimated parameters). For the special type of Beta function, the limit values of the *χ*^2^ test are *χ*^2^_0.025,32_ *=* 18.3 and *χ*^2^_0.975,32_ *=* 49.5, and for the Gamma and Weibull functions, these values are *χ*^2^_0.025,33_ *=* 19.0 and *χ*^2^_975,33_ *=* 50.7. For all of these functions the values of the *χ*^2^ statistic are outside of the defined intervals of the *χ*^2^ test. Analogous results were obtained for KC incidence rates in white females, as well as for PC incidence rates in both white males and white females (data not shown). Therefore, these results clearly show that the GB model has superior performance compared to the other considered models.

## Conclusion

In this work, we emphasized several general shortcomings in the mathematical modeling of age distribution of cancer, which include: the use of “raw” cancer data (inclusion of cases which were not microscopically confirmed or were not first primary cancers); the lack of consideration of time period and cohort effects on the observed incidence rates; and the omission of rigorous statistical evaluation of the determined model parameters. To overcome these shortcomings, we proposed a new approach, called the Generalized Beta (GB) model. This model utilizes observational data of age-specific incidence rates and uses sound statistical criteria to assess model parameters.

To test the performance of this model, we used “filtered” data from the SEER 9 database during the years of 1985–2004. We utilized these data to estimate the incidence rates of the first primary, microscopically confirmed cases of pancreatic cancer (PC) and kidney cancer (KC) in white males and females. These incidence rates were adjusted for time period and cohort effects. By the newly proposed GB approach, we approximated the adjusted incidence rates of the primary PC and KC in white males and females. Confidence intervals for model parameters were estimated by regression analysis. We showed that the age distributions of the KC and PC incidence rates have turnover points within the age interval of 74–81, after which these distributions fall off and reach the value of 0 (near the age of 100 years) at the end of the human life span.

The results presented in this work suggest that our approach significantly expands the possibilities of modeling of age distributions in PC and KC. We are certain that this approach could be generalized for many other organ-specific cancers and cancer subtypes and provide distinct model parameters that will be useful for the modeling of carcinogenic processes characteristic to particular cancers. It should be noted that in this work, we used the terms *degree of increase* or *degree of decrease* in a purely mathematical sense, because the precise mechanisms causing the increase and decrease of cancer incidence rates are not fully understood. To better understand the biological plausibility of the model parameters used in the proposed approach, detailed molecular, cellular and tissue-specific mechanisms underlying the development of each type of cancer will require further investigation. The model parameters that can be assessed by the proposed approach should challenge future biomedical and epidemiological studies.

## Figures and Tables

**Table 1:**
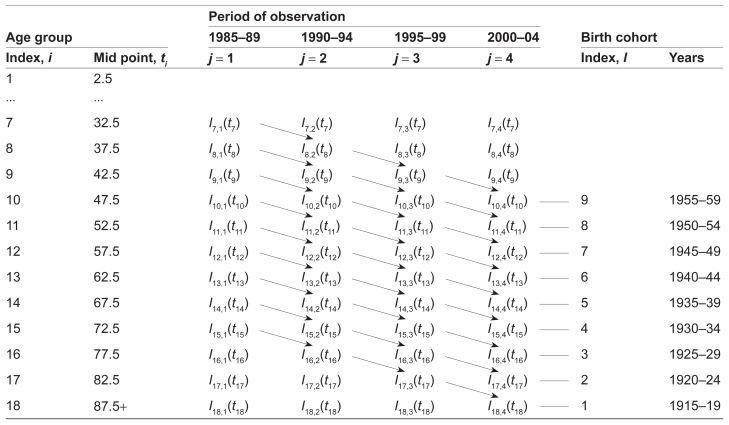
Presentation of the observed age-specific incidence rates for nine birth cohort groups during four time periods.

**Table 2:** Presentation of the observed incidence rates as the product of the hazard function, *h*(*t*), and the corresponding time period (*v*) and birth cohort (*u*) coefficients.

		Period of observation					
Age group		1985–89	1990–94	1995–99	2000–04	Birth cohort	
		
*Index, i*	*Mid point, t_i_*	*j* = 1	*j* = 2	*j* = 3	*j* = 4	Index, *l*	Years
1	2.5						
...	...						
7	32.5	*v*_1_*u*_9_*h*(*t*_7_)					
8	37.5	*v*_1_*u*_8_*h*(*t*_8_)	*v*_2_*u*_9_*h*(*t*_8_)				
9	42.5	*v*_1_*u*_7_*h*(*t*_9_)	*v*_2_*u*_8_*h*(*t*_9_)	*v*_3_*u*_9_*h*(*t*_9_)			
10	47.5	*v*_1_*u*_6_*h*(*t*_10_)	*v*_2_*u*_7_*h*(*t*_10_)	*v*_3_*u*_8_*h*(*t*_10_)	*v*_4_*u*_9_*h*(*t*_10_)	9	1955–59
11	52.5	*v*_1_*u*_5_*h*(*t*_11_)	*v_2_u_6_h*(*t*_11_)	*v_3_u_7_h*(*t*_11_)	*v*_4_*u*_8_*h*(*t*_11_)	8	1950–54
12	57.5	*v*_1_*u*_4_*h*(*t*_12_)	*v*_2_*u*_5_*h*(*t*_12_)	*v*_3_*u*_6_*h*(*t*_12_)	*v*_4_*u*_7_*h*(*t*_12_)	7	1945–49
13	62.5	*v*_1_*u*_3_*h*(*t*_13_)	*v*_2_*u*_4_*h*(*t*_13_)	*v*_3_*u*_5_*h*(*t*_13_)	*v*_4_*u*_6_*h*(*t*_13_)	6	1940–44
14	67.5	*v*_1_*u*_2_*h*(*t*_14_)	*v*_2_*u*_3_*h*(*t*_14_)	*v*_3_*u*_4_*h*(*t*_14_)	*v*_4_*u*_5_*h*(*t*_14_)	5	1935–39
15	72.5	*v*_1_*u*_1_*h*(*t*_15_)	*v*_2_*u*_2_*h*(*t*_15_)	*v*_3_*u*_3_*h*(*t*_15_)	*v*_4_*u*_4_*h*(*t*_15_)	4	1930–34
16	77.5		*v*_2_*u*_1_*h*(*t*_16_)	*v*_3_*u*_2_*h*(*t*_16_)	*v*_4_*u*_3_*h*(*t*_16_)	3	1925–29
17	82.5			*v*_3_*u*_1_*h*(*t*_17_)	*v*_4_*u*_2_*h*(*t*_17_)	2	1920–24
18	87.5+				*v*_4_*u*_1_*h*(*t*_18_)	1	1915–19

**Table 3:** *Age distribution of number of cases (shown as numerators) excluded from the total number of cases (shown as denominators) of KC and PC in white males and white females.* “Not first primary” denotes cases excluded due to secondary cancers or additional primary cancers. “Not confirmed” denotes cases excluded because the diagnosis was not microscopically confirmed by a pathologist. “Other” denotes cases excluded due to diagnosis by death certificate or autopsy record, in situ cancers, other non malignant tumors, and data records erroneously marked as first primary. Percentage of cases excluded is shown in parentheses.

	Male KC			Female KC		
Age	Not first primary	Not confirmed	Other	Not first primary	Not confirmed	Other
30–34	13/213 (6.1%)	4/213 (1.9%)	18/213 (8.5%)	11/168 (6.5%)	1/168 (0.6%)	12/168 (7.1%)
35–39	27/521 (5.2%)	3/521 (0.6%)	18/521 (3.5%)	21/313 (6.7%)	2/313 (0.6%)	10/313 (3.2%)
40–44	71/1074 (6.6%)	23/1074 (2.1%)	53/1074 (4.9%)	51/605 (8.4%)	10/605 (1.7%)	25/605 (4.1%)
45–49	125/1801 (6.9%)	43/1801 (2.4%)	67/1801 (3.7%)	108/824 (13.1%)	12/824 (1.5%)	37/824 (4.5%)
50–54	213/2424 (8.8%)	66/2424 (2.7%)	110/2424 (4.5%)	163/1251 (13.0%)	34/1251 (2.7%)	72/1251 (5.8%)
55–59	398/3112 (12.8%)	114/3112 (3.7%)	195/3112 (6.3%)	238/1576 (15.1%)	42/1576 (2.7%)	79/1576 (5.0%)
60–64	667/3738 (17.8%)	168/3738 (4.5%)	219/3738 (5.9%)	394/1876 (21.0%)	94/1876 (5.0%)	125/1876 (6.7%)
65–69	938/4013 (23.4%)	203/4013 (5.1%)	281/4013 (7.0%)	482/2321 (20.8%)	146/2321 (6.3%)	164/2321 (7.1%)
70–74	1116/4061 (27.5%)	285/4061 (7.0%)	371/4061 (9.1%)	537/2415 (22.2%)	234/2415 (9.7%)	194/2415 (8.0%)
75–79	1029/3359 (30.6%)	438/3359 (13.0%)	321/3359 (9.6%)	602/2396 (25.1%)	326/2396 (13.6%)	224/2396 (9.3%)
80–84	690/2088 (33.0%)	466/2088 (22.3%)	234/2088 (11.2%)	398/1726 (23.1%)	413/1726 (23.9%)	202/1726 (11.7%)

	Male PC			Female PC		
Age	Not first primary	Not confirmed	Other	Not first primary	Not confirmed	Other
30–34	2/64 (3.1%)	6/64 (9.4%)	3/64 (4.7%)	4/55 (7.3%)	1/55 (1.8%)	4/55 (7.3%)
35–39	11/186 (5.9%)	13/186 (7.0%)	6/186 (3.2%)	3/105 (2.9%)	2/105 (1.9%)	0/105 (0.0%)
40–44	12/410 (2.9%)	20/410 (4.9%)	12/410 (2.9%)	21/232 (9.1%)	9/232 (3.9%)	15/232 (6.5%)
45–49	40/776 (5.2%)	60/776 (7.7%)	22/776 (2.8%)	45/501 (9.0%)	21/501 (4.2%)	23/501 (4.6%)
50–54	68/1314 (5.2%)	117/1314 (8.9%)	30/1314 (2.3%)	85/843 (10.1%)	49/843 (5.8%)	28/843 (3.3%)
55–59	145/1826 (7.9%)	181/1826 (9.9%)	45/1826 (2.5%)	132/1292 (10.2%)	96/1292 (7.4%)	37/1292 (2.9%)
60–64	262/2497 (10.5%)	279/2497 (11.2%)	78/2497 (3.1%)	250/1792 (14.0%)	187/1792 (10.4%)	58/1792 (3.2%)
65–69	409/3139 (13.0%)	437/3139 (13.9%)	114/3139 (3.6%)	388/2742 (14.2%)	350/2742 (12.8%)	91/2742 (3.3%)
70–74	648/3556 (18.2%)	655/3556 (18.4%)	153/3556 (4.3%)	574/3491 (16.4%)	613/3491 (17.6%)	131/3491 (3.8%)
75–79	745/3243 (23.0%)	815/3243 (25.1%)	163/3243 (5.0%)	621/3871 (16.0%)	1044/3871 (27.0%)	223/3871 (5.8%)
80–84	563/2369 (23.8%)	886/2369 (37.4%)	153/2369 (6.5%)	588/3329 (17.7%)	1377/3329 (41.4%)	232/3329 (7.0%)

**Table 4:** Descriptive statistics and model parameters for pancreatic and kidney cancer.

	Pancreatic cancer		Kidney cancer	
	Male	Female	Male	Female
number of cancer cases (*n*)	13,919	13,306	20,054	12,106
Parameter *c ×* 10,000†	2.9 (2.1, 4.1)	1.5 (1.0, 2.4)	2.5 (1.9, 3.3)	0.3 (0.2, 0.5)
Parameter *k*†	10.2 (9.8, 10.5)	10.2 (9.8, 10.7)	8.9 (8.6, 9.1)	7.8 (7.4, 8.2)
Parameter *m*†	3.7 (3.6, 3.9)	3.4 (3.2, 3.6)	3.9 (3.8, 4.0)	3.2 (2.9, 3.4)
Weighted sum of residuals (R*_ν_)	25.0	39.0	24.5	46.0
Point (age) of inflection	64.3	66.7	58.6	60.6
Age of maximum incidence	77.0	79.1	72.8	75.7

† Numbers in parentheses denote 95% confidence intervals.
